# Supplementary material for: Hedgehog Signaling Pathway Orchestrates Human Lung Branching Morphogenesis
Source: Int J Mol Sci. 2022 May 9;23(9):5265. doi: 10.3390/ijms23095265 (PMC9100880; doi:10.3390/ijms23095265)
Supplement: Supplementary file 1 [file ijms-23-05265-s001.zip › ijms-1697127-supplementary.pdf]

Figure S1

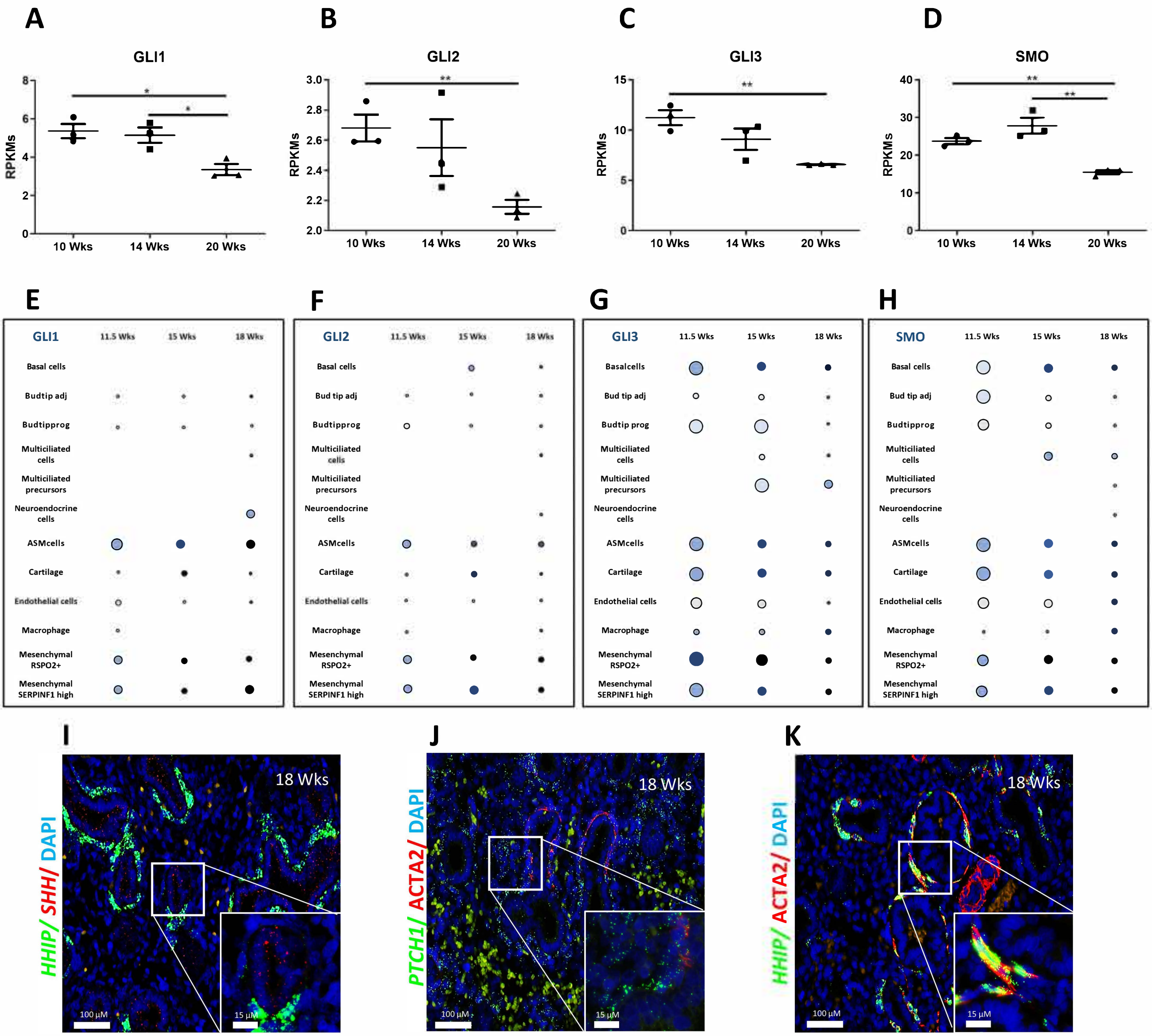

Figure S1: Complementary HH pathway actor expression during human lung development
RNA expression of *GLI1*(A), *GLI2* (B), *GLI3* (C) and *SMO* (D) expressed in RPKMs ± SEM within the developing lung at 10-, 14- and 20-week gestation (n=3 per time point, *p<0.05, p**<0.01). The dot plot shows the percentage of cells expressing the respective selected marker gene (*GLI1, GLI2, GLI3 and SMO*) using dot size and the average expression level of that gene based on unique molecular identifier (UMI) counts (E-H). Representative pictures of *in situ* hybridization at 18 wks gestation on fetal human lung sections for *SHH* (I, red), *PTCH1* (J, green) and HHIP (J-K, green) with ACTA2 (IF-red, J-K) and the respective dots quantification (L-N) (results show mean ±SEM, *p<0.05, n=3 for each group).

Figure S2

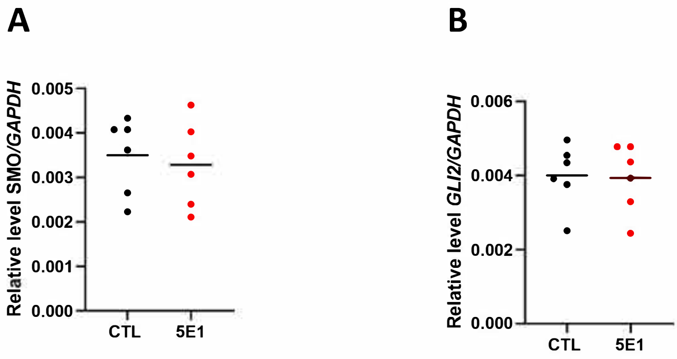

Figure S2: **Extended HH pathway components expression after 5E1 treatment**
RT-qPCR for *SMO* (A) and *GLI2* (B) in 5E1-treated explants compared to control (n=6 for each group).

Figure S3

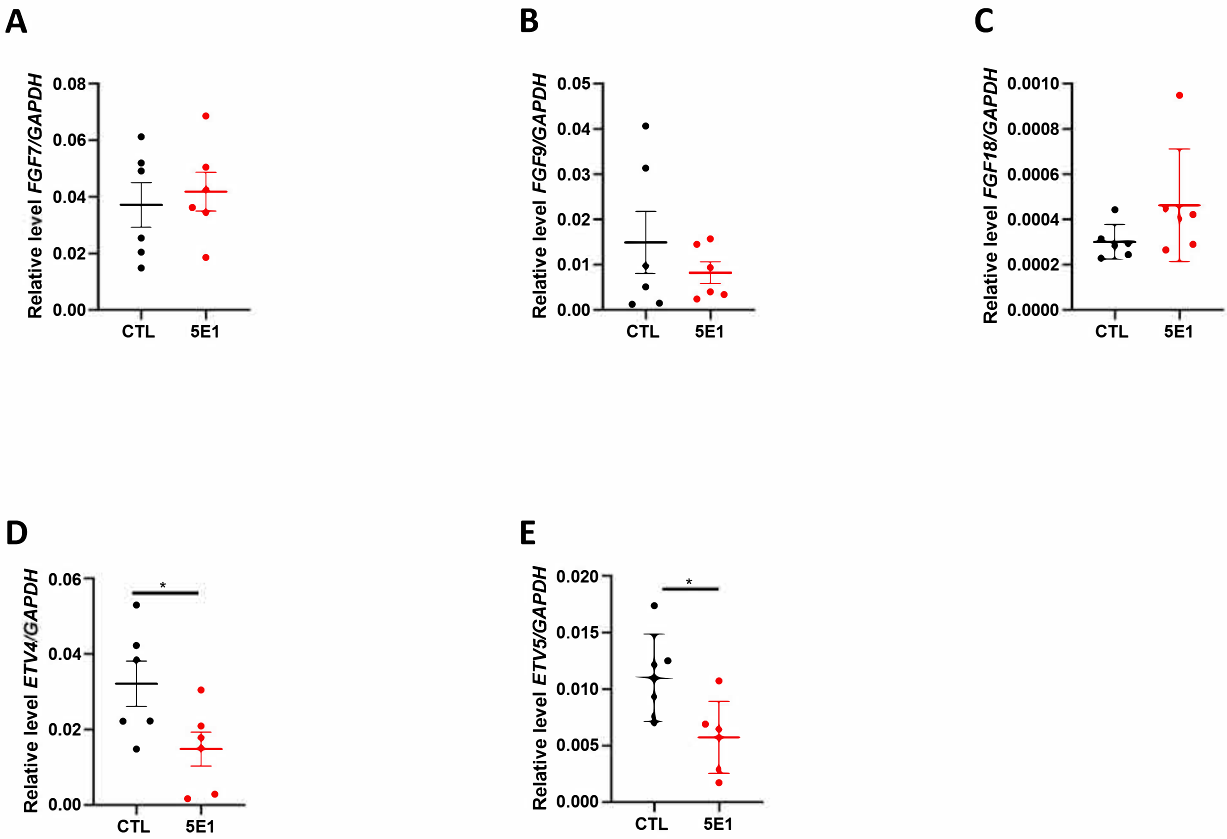

Figure S3: HH signaling effect on FGF and WNT pathway elements
RT-qPCR for *FGF7* (A), *FGF9* (B), *FGF18* (C), *ETV4* (D) and *ETV5* (E) in control and 5E1-treated explants (results show mean ±SEM, **p*<0.05, n=6 for each group).

Figure S4

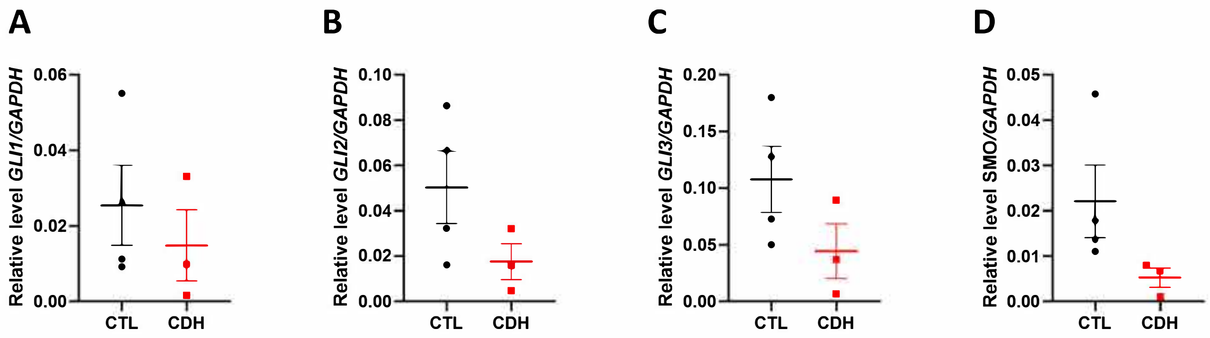

Figure S4: Additional HH pathway components expression in CDH

RT-qPCR for *GLI1* (A), *GLI2* (B), *GLI3* (C) and *SMO* (D) in 5E1-treated explants compared to controls.

Figure S5

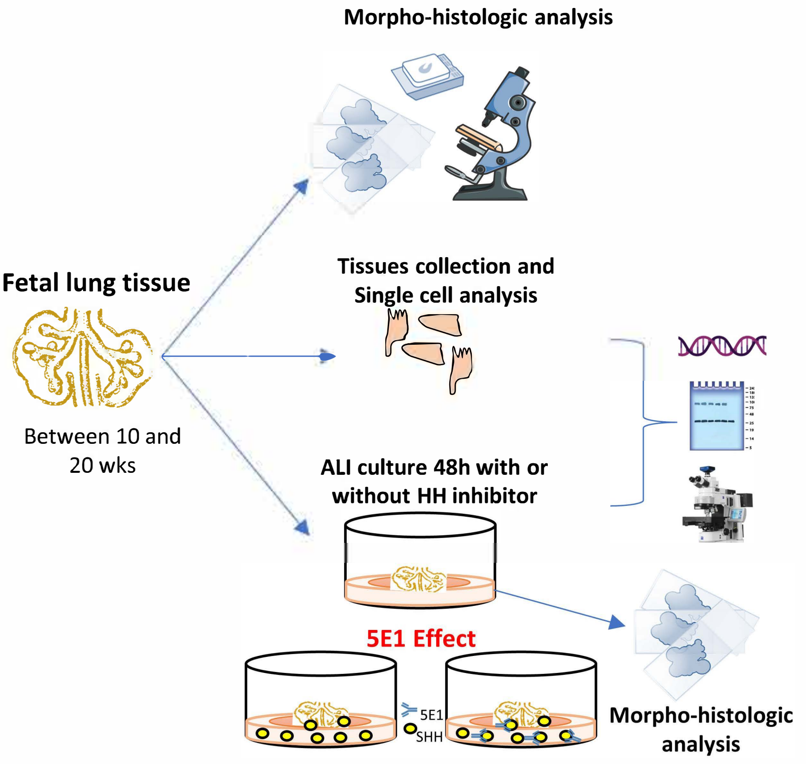

Figure S5: Schematic representation of methodology
Human fetal lung explant culture, from tissue to analyses.
